# Understanding Human-Virus Protein-Protein Interactions Using a Human Protein Complex-Based Analysis Framework

**DOI:** 10.1128/mSystems.00303-18

**Published:** 2019-04-09

**Authors:** Shiping Yang, Chen Fu, Xianyi Lian, Xiaobao Dong, Ziding Zhang

**Affiliations:** a State Key Laboratory of Agrobiotechnology, College of Biological Sciences, China Agricultural University, Beijing, China; b Department of Genetics, School of Basic Medical Sciences, Tianjin Medical University, Tianjin, China; Princeton University

**Keywords:** antiviral drug discovery, human-virus interaction, network, protein complex, protein-protein interaction

## Abstract

Computational analysis of human-virus protein-protein interaction (PPI) data is an effective way toward systems understanding the molecular mechanism of viral infection. Previous work has mainly focused on characterizing the global properties of viral targets within the entire human PPI network. In comparison, how viruses manipulate host local networks (e.g., human protein complexes) has been rarely addressed from a computational perspective. By mainly integrating information about human-virus PPIs, human protein complexes, and gene expression profiles, we performed a large-scale analysis of virally targeted complexes (VTCs) related to five common human-pathogenic viruses, including influenza A virus subtype H1N1, human immunodeficiency virus type 1, Epstein-Barr virus, human papillomavirus, and hepatitis C virus. We found that viral targets are enriched within human protein complexes. We observed in the context of VTCs that viral targets tended to have a high within-complex degree and to be scaffold and housekeeping proteins. Complexes that are essential for viral propagation were simultaneously targeted by multiple viruses. We characterized the periodic expression patterns of VTCs and provided the corresponding candidates that may be involved in the manipulation of the host cell cycle. As a potential application of the current analysis, we proposed a VTC-based antiviral drug target discovery strategy. Finally, we developed an online VTC-related platform known as VTcomplex (http://zzdlab.com/vtcomplex/index.php or http://systbio.cau.edu.cn/vtcomplex/index.php). We hope that the current analysis can provide new insights into the global landscape of human-virus PPIs at the VTC level and that the developed VTcomplex will become a vital resource for the community.

**IMPORTANCE** Although human protein complexes have been reported to be directly related to viral infection, previous studies have not systematically investigated human-virus PPIs from the perspective of human protein complexes. To the best of our knowledge, we have presented here the most comprehensive and in-depth analysis of human-virus PPIs in the context of VTCs. Our findings confirm that human protein complexes are heavily involved in viral infection. The observed preferences of virally targeted subunits within complexes reflect the mechanisms used by viruses to manipulate host protein complexes. The identified periodic expression patterns of the VTCs and the corresponding candidates could increase our understanding of how viruses manipulate the host cell cycle. Finally, our proposed conceptual application framework of VTCs and the developed VTcomplex could provide new hints to develop antiviral drugs for the clinical treatment of viral infections.

## Abstract (precis)

Although human protein complexes have been reported to be directly related to viral infection, previous studies have not systematically investigated human-virus PPIs from the perspective of human protein complexes. To the best of our knowledge, we have presented here the most comprehensive and in-depth analysis of human-virus PPIs in the context of VTCs. Our findings confirm that human protein complexes are heavily involved in viral infection. The observed preferences of virally targeted subunits within complexes reflect the mechanisms used by viruses to manipulate host protein complexes. The identified periodic expression patterns of the VTCs and the corresponding candidates could increase our understanding of how viruses manipulate the host cell cycle. Finally, our proposed conceptual application framework of VTCs and the developed VTcomplex could provide new hints to develop antiviral drugs for the clinical treatment of viral infections.

## INTRODUCTION

Due to its small genome, a virus has to take advantage of the established functions of human cellular proteins to complete its life cycle. Thus, an understanding of the molecular mechanisms involved in human-virus protein-protein interactions (PPIs) is very critical for the design of novel antiviral strategies. To date, numerous experimentally validated human-virus PPIs have been rapidly collected and compiled into multiple host-pathogen interaction databases. Of the multiple databases, HPIDB contains the most comprehensive human-virus PPI data by integrating multiple public resources (1). Based on the human-virus PPIs that have been identified, a series of computational analyses have been conducted (2–5). These studies have mainly focused on the global network attributes of viral targets within the entire human PPI network and have uncovered several important network patterns that govern human-virus interactions, such as the preferential targeting of host hubs and bottlenecks by viral proteins. In comparison, the local network properties of viral targets within the human PPI network have been addressed less comprehensively.

Proteins often assemble into complexes, also known as molecular machines, to perform a coordinated function. In general, a protein complex is comprised of multiple functionally diversified proteins (subunits) (6) and serves as a functional module or subnetwork within the entire PPI network (7). So far, a large number of protein complexes have been identified. The original CORUM database consisted of 622 human protein complexes that were systematically identified using proteomic profiling approaches (8). The updated version of CORUM (CORUM2.0) included 2,193 complexes that were comprised of approximately 3,200 proteins (9). Recently, a more comprehensive human protein complex data set named hu.MAP (10) has been released, which contains 4,659 complexes that are comprised of approximately 7,700 human proteins. The hu.MAP database integrated three recently published large-scale studies of human protein interaction data (11–13), which were generated from more than 9,000 mass spectrometry experiments, and used a machine learning method to reconstruct human protein complexes. These resources provide not only a global landscape of functional modules in the human proteome but also a reference map to characterize the role of each protein in the corresponding subnetwork.

Viral infection is tightly linked with host protein complexes. It has been established that viruses manipulate host protein complexes to regulate host biological processes (14–16). One example is the viral hijacking of the host eukaryotic initiation factor 4F complex to suppress gene expression in host cells, which ensures that viruses can maximally synthesize their own proteins and evade host immune responses (14). Another example is that viruses manipulate host complexes to accelerate or delay host cell cycle progression in order to favor their own replication as long as possible (17, 18). Indeed, for each human-virus PPI, the assignment of the corresponding virally targeted complex (VTC) is very informative in deciphering the functionality of the interspecies PPI. For instance, the function of the viral target could be inferred according to other members in this VTC. Currently, the functional roles of many human-virus PPIs remain elusive and await further clarification based on the investigation of VTCs (16). Therefore, the use of VTC-based analysis to increase the systematic understanding of viral infection is a pressing need.

Currently, the available data resources of human-virus PPIs and protein complexes have provided a golden computational opportunity for us to conduct systematic identification and analysis of VTCs. The discrimination of topological and functional properties of viral targets and nontargets within complexes could help us to understand how viruses manipulate host protein complexes. It is already known that a protein complex is a dynamic molecular machine, in which spatiotemporal regulation determines the subunit members and their corresponding functions within a cellular context (19). Therefore, the VTC-based analysis should also pay attention to the dynamic properties (e.g., temporal gene expression of subunits) of protein complexes, which has often been ignored in previous studies. Fortunately, projects such as GTEx (20) and time course RNA-Seq experiments (21) have measured the tissue-specific and dynamic expression of genes at the whole-genome level, which enables elucidation of how VTCs respond dynamically to viral infection.

In addition to providing an in-depth understanding of viral infection, research on human-virus PPIs is beneficial for antiviral drug development (22, 23). Current antiviral drugs mainly include virus- and host-targeting antiviral drugs (24). Virus-targeting drugs are designed to inhibit the biological function of viral proteins such as viral proteases and polymerases, while host-targeting drugs are able to disrupt the functioning of host proteins that are involved in the viral life cycle. Due to the rapid evolution of most viruses, resistance to virus-targeting drugs often causes failure in the clinic, especially for infections caused by RNA viruses. However, host-targeting drugs can reduce such effects because of the slower evolutionary rate of host proteins. Although some host-targeting drugs such as tromantadine (which targets human glycoproteins) and peginterferon alfa-2b (which targets human IFNARs) have already been used for the treatment of herpes simplex virus and hepatitis C virus (HCV), respectively (25), most known antiviral drugs target viral proteins (26). Therefore, it is urgent to identify druggable targets for developing new human-targeting antiviral drugs.

**FIG 1:**
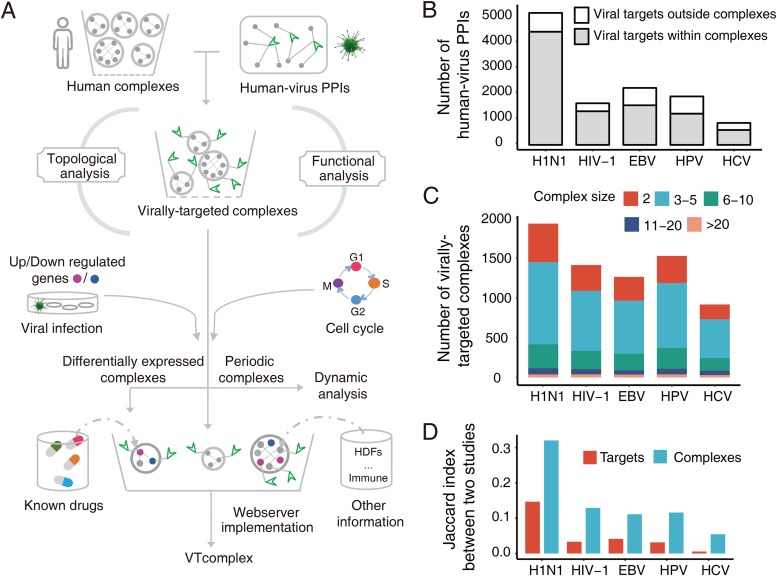
Landscape of VTCs related to the five viruses. (A) Flowchart of experimental design. (B) Numbers of human-virus PPIs with the corresponding targets within and outside complexes. (C) Sizes of the VTCs related to the five viruses. (D) Jaccard indices corresponding to the target and VTC level for each virus. The corresponding Jaccard index of each virus was calculated using data from two high-throughput human-virus PPI studies.

In this work, we revisited the investigation of human-virus PPIs at the VTC level. The overall flowchart that depicts our strategy is shown in Fig. 1A. First, we combined human-virus PPIs and human complex data to identify VTCs related to five viruses, including influenza A virus subtype H1N1 (H1N1), human immunodeficiency virus type 1 (HIV-1), Epstein-Barr virus (EBV), human papillomavirus (HPV), and HCV. Then, we analyzed the topological and functional attributes of viral targets within complexes. By mapping host gene expression data to VTCs, we analyzed the complexes responding to the viral infection and the dynamic properties of VTCs that are related to the host cell cycle. Moreover, we proposed a potential complex-based antiviral drug target discovery strategy by integrating human-virus PPIs, human protein complexes, and other heterogeneous information. Finally, a web portal was implemented for the community to access our identified VTCs and antiviral drug discovery-related information.

## RESULTS

### The landscape of human protein complexes targeted by five viruses.

The current analysis of VTCs was based on hu.MAP, since it is the most comprehensive data resource for human protein complexes. We first collected 4,589 human protein complexes from hu.MAP (10) and 11,846 human-virus PPIs from HPIDB (1). For H1N1, HIV-1, EBV, HPV, and HCV, the numbers of human-virus PPIs are 5,195, 1,638, 2,242, 1,907, and 864, respectively (Fig. 1B; see also Data Set S1 in the supplemental material). To identify VTCs, we considered only human-virus PPIs whose viral targets are contained within protein complexes. The results showed that 77.2% (9,147/11,846) of the human-virus PPIs, which covered 69.6% (2,755/3,961) of the viral targets (Fig. 1B; see also Fig. S1A in the supplemental material), could be used for further analysis. For each virus under investigation, we further integrated human-virus PPIs and human protein complexes to identify the corresponding VTCs. VTCs were defined as protein complexes that contained at least one subunit targeted by viral proteins. For example, a complex that was considered to be targeted by HIV-1 means that at least one protein within the complex interacts with HIV-1 proteins. We observed that viruses significantly targeted human protein complexes (hypergeometric test, *P* value < 2.2 × 10^−16^; Fig. S1B). This demonstrated that protein complexes are highly involved in viral infection. After mapping 9,147 human-virus PPIs to 4,589 protein complexes, we identified 3,139 nonredundant VTCs in total, and the corresponding numbers of VTCs related to H1N1, HIV-1, EBV, HPV, and HCV are 1,930, 1,411, 1,262, 1,525, and 916, respectively (Fig. 1C and Data Set S2). The overlap of VTCs among the different viruses is shown in Fig. S2A. To further quantify the similarity between viruses in terms of targeting human protein complexes, the Jaccard indices determined using the comparison of VTCs related to any two viruses are also shown in Fig. S2B. Note that the Jaccard index was calculated by dividing the number of VTCs related to both viruses by the number of VTCs related to either of the two viruses. We found that H1N1 and HIV-1 had the largest Jaccard index (0.41), which is likely because both H1N1 and HIV-1 are RNA viruses.

10.1128/mSystems.00303-18.2 FIG S1 Relationship between viral targets and subunits in complexes. (A) Numbers of viral targets within and outside protein complexes for five viruses. (B) Venn diagram showing the overlap between viral targets and subunits in all complexes. Three asterisks indicate a *P* value of <0.001. Download FIG S1, PDF file, 0.8 MB. Copyright © 2019 Yang et al. 2019 Yang et al. This content is distributed under the terms of the Creative Commons Attribution 4.0 International license.

10.1128/mSystems.00303-18.3 FIG S2 Overlap of all VTCs among five viruses. (A) Venn diagram showing the number of overlapping complexes among five viruses. (B) Jaccard indices showing the similarities of VTCs between any two viruses. (C) Proportions of subunits within six complexes targeted by a different number of viruses. These six complexes are significantly targeted by five viruses (VT_significance_ < 0.05). Download FIG S2, PDF file, 0.9 MB. Copyright © 2019 Yang et al. 2019 Yang et al. This content is distributed under the terms of the Creative Commons Attribution 4.0 International license.

10.1128/mSystems.00303-18.8 DATA SET S1 List of human-virus PPIs. Download Data Set S1, XLSX file, 0.3 MB. Copyright © 2019 Yang et al. 2019 Yang et al. This content is distributed under the terms of the Creative Commons Attribution 4.0 International license.

10.1128/mSystems.00303-18.9 DATA SET S2 List of VTCs related to five viruses and annotation information of VTCs. Download Data Set S2, XLSX file, 2.2 MB. Copyright © 2019 Yang et al. 2019 Yang et al. This content is distributed under the terms of the Creative Commons Attribution 4.0 International license.

Previous studies have shown a limited overlap between large-scale interspecies PPIs using identification experiments conducted with the same human-virus system (27, 28). Here we used human-virus PPIs identified based on different independent studies to determine the overlap of the experimental results at the VTC level using the Jaccard index (Fig. 1D and Table S1). Note that the Jaccard index was calculated by dividing the number of VTCs identified in both independent studies by the number of VTCs identified in either of the two independent studies. The results showed that the Jaccard index for the VTCs ranged from 0.05 to 0.32 in all five viruses, which was at least double the corresponding value found for the targets; this confirmed the rational basis and usefulness of conducting large-scale VTC analysis.

10.1128/mSystems.00303-18.1 TABLE S1 PubMed IDs used for counting the Jaccard index at the target/VTC level. Download Table S1, DOCX file, 0.03 MB. Copyright © 2019 Yang et al. 2019 Yang et al. This content is distributed under the terms of the Creative Commons Attribution 4.0 International license.

### Topological and functional analyses of human protein complexes targeted by viruses.

**FIG 2:**
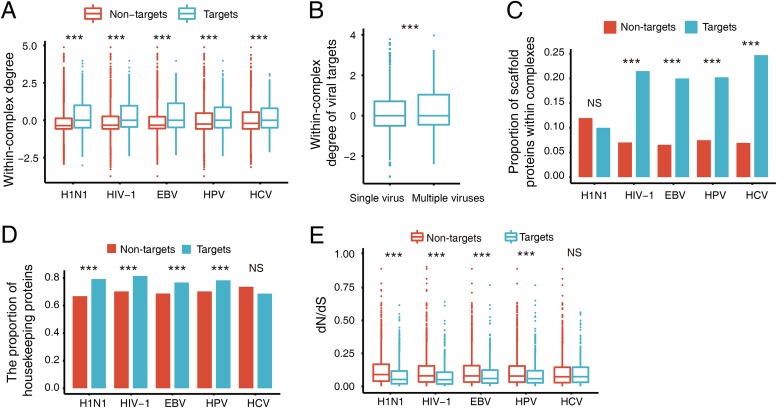
Comparison of the topological and functional characteristics between viral targets and nontargets within VTCs. (A) Within-complex degree distributions of the proteins. (B) Within-complex degree distributions of the proteins targeted by single and multiple viruses. (C) Proportions of scaffold proteins. (D) Proportions of housekeeping proteins. (E) d_N_/d_S_ distribution of the proteins. Values that are significantly different (*P* < 0.001) are indicated by three asterisks. Values that are not significantly different (*P* > 0.05) are indicated (NS). The *P* values in panels A, B, and E were calculated by one-tailed Wilcoxon rank sum test, and the *P* values in panels C and D were calculated by one-tailed two-proportion z-test.

In the context of network biology, an efficient way for viruses to manipulate host cells is to target the hubs of host PPI networks. To examine the relevant network patterns in subnetworks such as VTCs, we mapped experimentally validated human PPIs to VTCs and calculated the within-complex degree of each subunit in the VTCs. Note that the within-complex degree is defined as the relative degree of a protein in the corresponding complex (see Materials and Methods for the calculation of the within-complex degree). We found that the within-complex degree of viral targets was significantly higher than that of nontargets (Wilcoxon rank sum test, *P* value < 2.2 × 10^−16^ for H1N1, HIV-1, EBV, and HPV, and *P* value = 3.7 × 10^−6^ for HCV; Fig. 2A). Moreover, we observed that the within-complex degrees of subunits targeted by multiple viruses were significantly higher than those of subunits targeted only by a single virus (Wilcoxon rank sum test, *P* value < 2.2 × 10^−16^; Fig. 2B).

We also examined the functionality and conservation of viral targets in VTCs. First, we focused on scaffold proteins, which are an important type of protein that provide scaffolds to promote the assembly of protein complexes and thus play crucial roles in cellular signaling pathways (29). We compared the proportions of scaffold proteins in targets and nontargets within VTCs. Here we considered only VTCs containing at least one scaffold protein. The results showed that four of the five viruses had a significantly higher proportion of scaffold proteins in targets compared to nontargets (one-tailed two-proportion z-test, *P* value of 2.8 × 10^−16^ for HIV-1, 2.8 × 10^−13^ for EBV, 8.9 × 10^−12^ for HPV, and 7.8 × 10^−14^ for HCV; Fig. 2C). Note that this was not observed for H1N1 (*P* value = 0.87; Fig. 2C). This indicated that viral proteins generally tended to target scaffold proteins and thereby hamper the correct assembly of host protein complexes. Next, we focused on the distribution of housekeeping genes within VTCs. Housekeeping genes are those genes expressed in all cells of an organism, which encode key proteins maintaining basic cellular life (30). We classified the protein subunits as housekeeping proteins or nonhousekeeping proteins. The results showed that viral targets significantly tended to be housekeeping proteins compared to nontargets within VTCs (one-tailed two-proportion z-test, *P* value < 2.2 × 10^−16^ for H1N1 and HIV-1, 2.46 × 10^−11^ for EBV, and 1.25 × 10^−13^ for HPV; Fig. 2D). The only exception was HCV (*P* value = 0.99, Fig. 2D), which may be due to the limited number of VTCs detected in human-HCV interaction and/or the different biological mechanisms adopted by HCV. The preference for targeting housekeeping proteins allows viruses to be more efficient in manipulating the corresponding complexes. This phenomenon was further confirmed by the examination of the evolutionary conservation of viral targets. By introducing the calculation of d_N_/d_S_, which is the ratio of nonsynonymous to synonymous mutations, we observed that viral targets showed a higher level of evolutionary conservation (Wilcoxon rank sum test, *P* value < 2.2 × 10^−16^ for H1N1 and HIV-1, *P* value = 4.6 × 10^−8^ for EBV, *P* value = 3.5 × 10^−8^ for HPV, and *P* value = 0.95 for HCV; Fig. 2E).

On the basis of the above analyses of targets and nontargets within complexes, we noticed a data imbalance in the human-virus PPIs among the different viruses. For instance, the number of human-H1N1 PPIs is approximately six times larger than that of human-HCV PPIs. To determine the influence of human-virus PPI data coverage, we performed a down-sampling of the human-H1N1 PPIs and then repeated the above analysis. Specifically, we randomly selected 864 pairs of human-H1N1 PPIs (which is the same number of human-HCV PPIs used in this work) and reanalyzed the preference of H1N1 for targeting housekeeping proteins within complexes as an example to determine the influence of data coverage. The random trial was repeated 1,000 times. The results verified that H1N1 tends to target housekeeping proteins within complexes (empirical *P* value = 0.004). Therefore, we concluded that our findings are generally robust even when there are variations in data coverage; however, we cannot directly rule out any influence of data coverage on the human-HCV PPI analysis.

**FIG 3:**
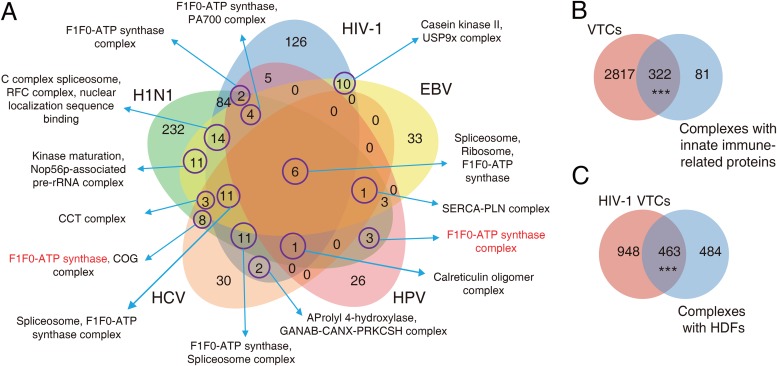
Functional annotations of the VTCs. (A) Venn diagram shows the number of common and specific VTCs (VT_significance_ < 0.05). Representative complex names are labeled; an example of VTCs with similar functions but that are comprised of different subunits is shown in red. (B) Overlap between all viruses’ VTCs and complexes containing innate immune-related proteins. (C) Overlap between HIV-1 VTCs and complexes containing HDFs. Three asterisks denote a *P* value of <0.001, and NS (not significantly different) denotes a *P* value of >0.05.

Subsequently, we focused on the functional distribution of the VTCs. To effectively prioritize the VTCs for each virus, we defined a scoring variable known as VT_significance_ to measure the significance of a complex targeted by a specific virus. More details regarding the calculation of VT_significance_ are available in Materials and Methods. The overlap among complexes significantly targeted by each of the five viruses (VT_significance_ < 0.05) is shown in Fig. 3A. We observed that six complexes, including spliceosome, ribosome, and the F1F0-ATP synthase complex (Fig. 3A and Fig. S2C) were targeted by all five viruses, which is consistent with the functional importance of these complexes to viral propagation (31). The spliceosome is an important complex involved in host transcriptional regulation (32), the ribosome acts as translational machinery that can be manipulated by viruses to synthesize proteins for their replication (14), and the F1F0-ATP synthase complex acts as a metameric protein complex that is essential for ATP synthesis which can be used to provide energy for viral replication. We further investigated two classes of proteins named innate immune proteins and host dependency factors (HDFs) because these proteins have been shown to be highly related to viral infection (33, 34). Innate immune-related proteins play an important role in the detection of viral invasion. The results showed that the VTCs of all five viruses had a significant overlap with complexes containing innate immune proteins (hypergeometric test, *P* value = 1.1 × 10^−11^; Fig. 3B), which revealed the involvement of these protein complexes in host antiviral defense. Viruses depend on a series of HDFs for their replication. Here, we took only the VTCs related to HIV-1 into account, since the collected HDFs were HIV-1 specific. Similarly, complexes containing HDFs had a significant overlap with the VTCs of HIV-1 (hypergeometric test, *P* value < 2.2 × 10^−16^; Fig. 3C), which indicated that some of these complexes may be essential for HIV-1 infection.

### Dynamic response of human protein complexes targeted by viruses during viral infection.

**FIG 4:**
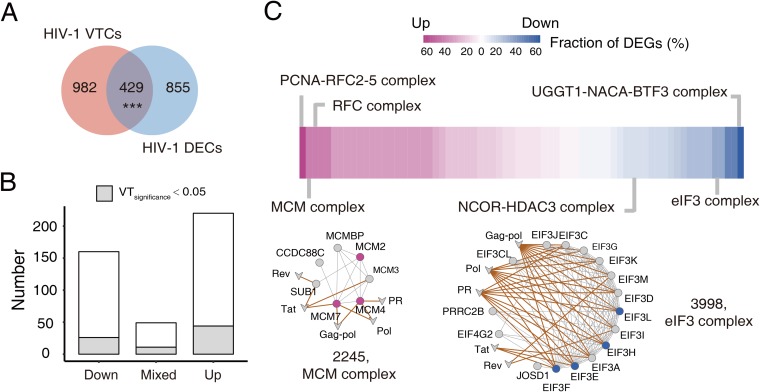
VTCs responding to viral infection. (A) Overlap between HIV-1 VTCs and DECs. (B) Numbers of different classes of HIV-1-targeted DECs. The gray region represents DECs with a VT_significance_ of <0.05. (C) Selected HIV-1 significantly targeted DECs (VT_significance_ < 0.05). A total of 81 upregulated or downregulated DECs are ranked according to the fraction of DEGs they contain. In addition, network representations of two DECs are provided. Nodes in purple represent upregulated genes, while nodes in blue represent downregulated genes. Circles represent human proteins, and V shapes represent viral proteins.

To investigate the dynamic response of VTCs to viral infection, analysis of changes in the host transcriptome resulting from viral infection was conducted. We conducted this analysis only for HIV-1, but it could be extended to other viruses. We first collected known differentially expressed genes (DEGs) during HIV-1 infection from the study by Sherrill-Mix et al. (35), in which gene expression changes in T cells 48 h after HIV-1 infection were measured. A complex containing at least one DEG was defined as a differentially expressed complex (DEC). Thus, 1,284 DECs were identified (Data Set S3). Then, we calculated the overlap between the DECs and VTCs. The results showed that the VTCs significantly overlapped with the DECs (hypergeometric test, *P* value = 6.9 × 10^−3^; Fig. 4A). We then focused on analysis of the overlapping complexes. To distinguish their different responses to viral infection, we classified the DECs into three classes: Down (all downregulated DEGs), Up (all upregulated DEGs), and Mixed (simultaneously down- and upregulated DEGs). It was revealed that most of the complexes had concordant responses to viral infection, and the proportion of DECs in the Mixed class is only 11.1% (Fig. 4B).

10.1128/mSystems.00303-18.10 DATA SET S3 Lists of HIV-1-targeted DECs and all periodic complexes. Download Data Set S3, XLSX file, 0.07 MB. Copyright © 2019 Yang et al. 2019 Yang et al. This content is distributed under the terms of the Creative Commons Attribution 4.0 International license.

The biological significance of the above analysis was further exemplified in two representative complexes (Fig. 4C). We found that the minichromosome maintenance (MCM) complex was upregulated. The MCM complex plays an important role in the control of host DNA replication as well as viral genome replication. A previous study has shown that replication of the RNA genome of the influenza virus is decreased in MCM component 2 (MCM2) knockdown cells (36), implying that the upregulation of MCM favors replication of the HIV-1 genome. Another representative complex, the eukaryotic initiation factor 3 (eIF3) complex was shown to be downregulated. The eIF3 complex plays a vital role in the initiation stage of eukaryotic translation. A possible explanation for the downregulation of eIF3 is that HIV-1 inhibits the expression of eIF3 to reduce host protein expression for the purpose of transferring more resources for its own replication. Taken together, the integration of differential expression information into complexes has provided an in-depth understanding of the functional importance of VTCs related to viral infection.

### Temporal mapping of human protein complexes targeted by viruses during the host cell cycle.

**FIG 5:**
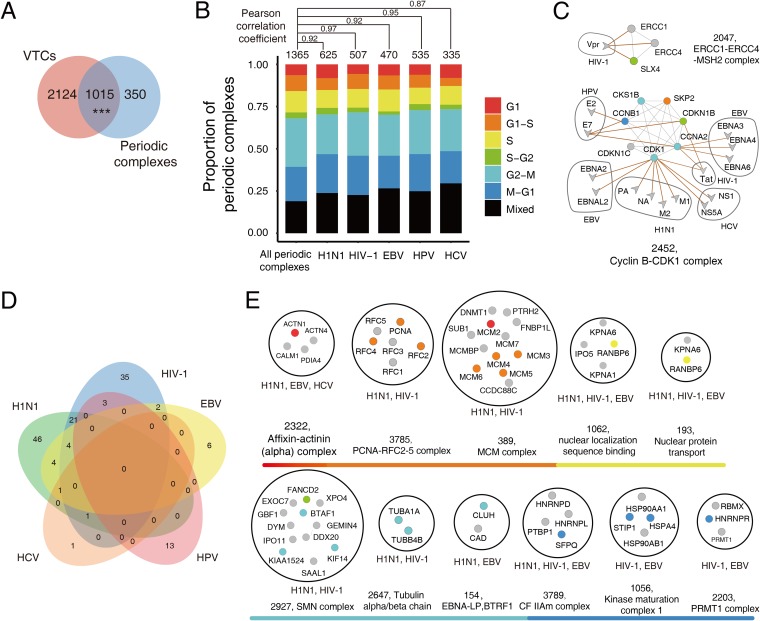
Temporal mapping of VTCs during the host cell cycle. (A) Overlap between VTCs and periodic complexes. (B) Proportions of virally targeted periodic complexes at different cell cycle phases. The colors indicate different cell cycle phases. For comparison, all periodic complexes were used as the background, and the PCC values between the proportions of virally targeted periodic complexes and the periodic complexes at different cell cycle phases were labeled for each virus. (C) Network representation of two experimentally validated periodic complexes involved in the manipulation of the host cell cycle. (D) Overlap among viral significantly targeted periodic complexes (VT_significance_ < 0.05 and Periodic_ratio_ > 0.2) related to five viruses. (E) Periodic complexes targeted by multiple viruses during the G_1_, G_1_-S, S, S-G_2_, G_2_-M, or M-G_1_ phase (VT_significance_ < 0.05 and Periodic_ratio_ > 0.2). The color scheme of nodes in panel B is also used in panels C and E.

During the cell cycle in human cells, most protein complexes are assembled “just in time,” in which periodic proteins that are vital for the assembly and functionality of certain complexes are expressed at their highest level during a specific phase (37). Viruses have been reported to arrest the host cell cycle at specific phases by hijacking host protein complexes. Therefore, to study how viruses manipulate the host cell cycle, we need to determine which periodic complexes were targeted by viruses during different cell cycle phases. We first mapped 1,221 known periodic proteins into all protein complexes to identify all of the periodic complexes, which resulted in the identification of 1,365 periodic complexes (see Materials and Methods for more details about the identification of the periodic proteins/complexes). The overlap between the VTCs and periodic complexes was significant (hypergeometric test, *P* value = 4.6 × 10^−9^; Fig. 5A), which indicated that viruses were involved in the assembly of protein complexes during the host cell cycle. We then classified these virally targeted periodic complexes into groups based on specific cell cycle phases (G_1_, G_1_-S, S, S-G_2_, G_2_-M, and M-G_1_), as well as a Mixed group, which contained periodic subunits that were expressed in more than one cell cycle phase, in order to determine whether different viruses tend to target complexes at specific cell cycle phases. We also calculated the Pearson correlation coefficient (PCC) between the proportions of virally targeted periodic complexes and periodic complexes at different cell cycle phases for each virus. Overall, the proportion of virally targeted periodic complexes is similar to that of the periodic complexes expressed at different cell cycle phases (average PCC = 0.93; Fig. 5B). Moreover, we calculated the proportions of periodic targets and nontargets within complexes during different cell cycle phases for each virus, and then the PCC values of the proportion of periodic proteins (for periodic targets and nontargets, respectively) between any two viruses were obtained (Fig. S3). Interestingly, we found that the proportion was more conserved in periodic nontargets (average PCC = 0.98) than in periodic targets (average PCC = 0.84), which indicated that different viruses tended to arrest phase-specific subunits when targeting the same periodic complex (Fig. 5B and Fig. S3). Among those virally targeted periodic complexes, note that experimentally validated host cell cycle arrest-related complexes such as SLX4 (complex ID = 2047) (38) and cyclin B-CDK1 (complex ID = 2452) (39, 40) were successfully identified (Fig. 5C).

10.1128/mSystems.00303-18.4 FIG S3 Proportions of periodic proteins within virally targeted periodic complexes in different periodic phases. The periodic proteins include targets and nontargets. Download FIG S3, PDF file, 0.9 MB. Copyright © 2019 Yang et al. 2019 Yang et al. This content is distributed under the terms of the Creative Commons Attribution 4.0 International license.

Currently, most of the known cell cycle arrest-related complexes are associated with cyclin-dependent kinases (CDKs), and the periodic phases that have been reported to be subverted by viruses are involved mainly in cell cycle checkpoints. Considering that regulation of the cell cycle is quite complicated, identification of periodic complexes that are involved in viral infection is far from complete. To identify other potential cell cycle-related protein complexes, Periodic_ratio_ was defined to measure the degree of dynamics within a complex, which was equal to the number of periodic proteins divided by the number of all subunits in the complex. A larger Periodic_ratio_ value indicates that a complex is more dynamic. We selected a subset of highly dynamic periodic VTCs (for which VT_significance_ < 0.05 and Periodic_ratio_ > 0.2) to generate a time series map of VTCs sorted by cell cycle phases. Detailed information about the highly dynamic periodic complexes that are targeted during each phase by five viruses are shown in Fig. S4. In addition, the overlaps among the periodic complexes targeted by the five viruses are shown in Fig. 5D. Overall, the functions of the periodic complexes are consistent with the biological events that occur in the corresponding phases. For example, the G_1_-S transition is considered a major cell cycle checkpoint, during which the host cell verifies DNA integrity. Among the identified periodic complexes that corresponded to this phase, DNA repair complexes such as MSH2-MSH6 (complex ID = 2620) and MSH2-RECQL complex (complex ID = 167) were shown to be targeted by HIV-1 (Fig. S4). Finally, we focused on periodic complexes targeted by multiple viruses because these complexes may reveal common infection patterns used by viruses to commandeer the periodic complexes of human cells. Of the identified 36 complexes targeted by multiple viruses, 11 complexes were distributed within specific phases (Fig. 5E), while the other 25 complexes contained mixed periodic subunits (Data Set S3). Collectively, through temporal mapping of periodic information to the VTCs, we obtained an elaborate map of the VTCs involved in different cell cycle phases of the entire cell cycle.

10.1128/mSystems.00303-18.5 FIG S4 Virally targeted periodic complexes in different periodic phases. Selected virally targeted periodic complexes (VT_significance_ < 0.05 and Periodic_ratio_ >0.2) were classified into six groups based on specific cell cycle phases (G_1_, G_1_-S, S, S-G_2_, G_2_-M, and M-G_1_). Circles represent human proteins, and V shapes represent viral proteins. Nodes in different colors represent periodic proteins in different phases. Edges in brown represent human-virus interactions. Download FIG S4, PDF file, 1.4 MB. Copyright © 2019 Yang et al. 2019 Yang et al. This content is distributed under the terms of the Creative Commons Attribution 4.0 International license.

### Discovery of potential antiviral drug targets based on human protein complexes targeted by HIV-1.

Because a protein complex is a complete functional unit, disruption of the expression levels of its subunits may have dramatic effects on complex formation (41). Drugs that perform their function by disturbing the formation of protein complexes have been reported (42). Inspired by previous studies, we attempted here to seek potential antiviral drug targets based on VTCs. We selected HIV-1 as a case study to show how we combined heterogeneous information for the discovery of potential antiviral targets.

**FIG 6:**
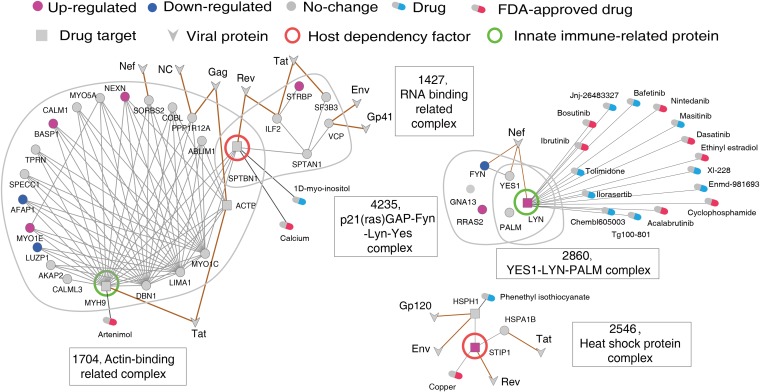
Network representation of the identified anti-HIV-1 druggable complexes as well as the potential druggable targets and drugs. For the associated subnetworks (VTCs), the corresponding complex IDs and names are shown. Circles represent human proteins, V shapes represent viral proteins, and squares represent druggable targets. Nodes in purple represent upregulated genes, while nodes in blue represent downregulated genes.

Our antiviral drug target discovery approach is based on the host protein complex. The first step was to identify antiviral druggable complexes according to the following criteria. (i) The complex is a VTC. (ii) The complex is a DEC. (iii) The complex contains at least one known drug target that is specifically expressed in virally targeted tissues and is not an essential gene. Using these criteria, we mapped expression data obtained during HIV-1 infection of VTCs and identified 61 HIV-1-targeted DECs (for which VT_significance_ < 0.05 and DEG_ratio_ > 0.1). Note that the DEG_ratio_ reflects the extent of a complex’s response to viral infection and is calculated by dividing the number of DEGs by the total number of subunits in the complex. The distribution of DEG_ratio_ values for all HIV-1-targeted DECs is shown in Fig. S5. We then tried to identify the druggable subunits within these 61 DECs. We further selected complexes with at least one known drug target in DrugBank (43) or TTD (44). Moreover, these drug targets should be nonessential genes and should have higher expression in HIV-1-infected tissues (DT_specificity_ > 2; see Materials and Methods for the definition) to ensure their correlation with viral infections. Finally, we identified 16 druggable complexes with 19 drug targets. Previous studies have already demonstrated that HDFs and immune-related proteins could be used as antiviral drug targets (24, 45, 46). To further narrow down the number of druggable complex candidates, five complexes containing HDFs or immune-related proteins were selected, including the RNA-binding-related complex (complex ID = 1427), actin-binding-related complex (complex ID = 1704), p21(ras)GAP-Fyn-Lyn-Yes complex (complex ID = 4235), Yes1-Lyn-PALM complex (complex ID = 2860), and heat shock protein complex (complex ID = 2546) (Fig. 6). We anticipated that these five complexes could serve as potential antiviral druggable complexes.

10.1128/mSystems.00303-18.6 FIG S5 Distribution of DEG_ratio_ values for all HIV-1-targeted DECs. The DEG_ratio_ ranges from 0 to 1. Download FIG S5, PDF file, 0.7 MB. Copyright © 2019 Yang et al. 2019 Yang et al. This content is distributed under the terms of the Creative Commons Attribution 4.0 International license.

Among these five druggable complexes, there were four known drug targets, including two HDFs (SPTBN1 and STIP1), and two immune-related proteins (LYN and MYH9). We proposed that these four proteins should be utilized preferentially as potential antiviral drug targets. Following the strategy of drug repurposing, we subsequently attempted to identify potential antiviral drugs based on known drugs. By mapping known drugs in DrugBank or DGIdb (47) to these four drug targets, we identified 21 drugs interacting with these drug targets (Fig. 6). To further clarify the proposed strategy of antiviral drug target discovery, the target MYH9 was exemplified. As a key subunit in the actin-binding-related complex, MYH9 plays a role in cytokinesis and frequently interacts with others subunits. Interestingly, we found that the known drug artenimol, which is used in the treatment of uncomplicated Plasmodium falciparum infections, inhibits MYH9. On the basis of our predictions, artenimol may also serve as a potential antiviral drug. Overall, we demonstrated how we integrated heterogeneous information to predict potential drug targets; however, the reliability of these predicted drug targets and drugs should be explored via further experimental validation.

### Development of an online platform for human protein complexes targeted by viruses.

To facilitate the community, we also built an online platform named VTcomplex to collect and manage VTC-related information, including the known human-virus PPIs for five viruses, human protein complexes, human intraspecies PPIs, potential drug-target interactions, and other key protein information related to viral infection. VTcomplex supports multiple searching keywords, including protein/gene name, UniProt ID, and complex name/ID. Regarding the webpage of a specific VTC, the intraspecies and interspecies PPIs are visualized in network format on the left panel, while other heterogeneous information such as HDFs, innate immune-related genes, and dynamic information about the subunits (DEG and periodic expression data) within this complex is shown on the right panel. An example of a query for HIV-1 Vif protein targeted complexes is shown in Fig. S6. As this is the first online resource for VTCs, we anticipate that VTcomplex will strengthen our in-depth understanding of human-virus interactions as well as provide some new hints regarding antiviral drug discovery.

10.1128/mSystems.00303-18.7 FIG S6 Flowchart for using VTcomplex. Download FIG S6, PDF file, 2.2 MB. Copyright © 2019 Yang et al. 2019 Yang et al. This content is distributed under the terms of the Creative Commons Attribution 4.0 International license.

## DISCUSSION

In this work, through integrating information about human-virus PPIs and human complexes, we conducted a large-scale computational identification and systematic analysis of VTCs. Previous studies regarding the identification and analysis of VTCs often concentrated on an individual virus or several specific VTCs. Thus, the number of VTCs investigated in previous studies has been very limited. For instance, a recent study of VTCs targeted by HIV analyzed only 40 complexes (31). Thanks to the rapid increase in the number of human-virus PPIs and human protein complexes that have been deposited in public databases, our large-scale analysis identified all of the potential VTCs for five common viruses. We also defined the scoring variable VT_significance_ to effectively prioritize the VTCs for each virus. Moreover, we constructed a web portal called VTcomplex to display all relevant information in the form of text and graphics, which will be helpful in obtaining a quick overview of each VTC. To our best knowledge, the developed VTcomplex is the first online platform for VTCs.

The current VTC-based analysis has allowed us to understand human-virus interactions from a unique perspective, which is different from previous analyses based on the entire human PPI network. With regards to network analysis, we focused on the within-complex topological features. VTCs should be regarded as subnetworks in the context of the entire human PPI network. The observation that viruses target human proteins with higher degrees in a complex is consistent with results that have been inferred from the entire network. However, the potential biological meaning may be different. Targeting hubs within the entire network may influence multiple processes, while targeting within-complex hubs may intensively disturb protein complexes. We observed that at the subunit level, viral targets tend to be functionally important proteins, such as housekeeping proteins and scaffold proteins. The topological and functional differences between viral targets and nontargets clearly revealed viral targeting preferences of subunits in VTCs. We found that at the complex level, multiple viruses significantly target complexes related to basic viral self-reproduction, which allows us to catch a glimpse of the common infection mechanisms. Indeed, complexes related to transcription and translation regulation and energy production are significantly targeted by all five viruses. Interestingly, we also found that some virus-specific complexes have similar functions (Fig. 3A), which suggests that viruses manipulate common biological processes by targeting functionally related host protein complexes in a virus-specific manner. For instance, we observed that two different F1F0-ATP synthase-related complexes (complex IDs = 128 and 3822) are targeted by HCV and HPV, respectively, although the subunits they contain are not exactly the same.

To characterize the dynamic properties of VTCs during the host cell cycle, we conducted a temporal mapping of VTCs into different periodic phases. The dynamic properties of the VTCs revealed common periodic complexes manipulated by different viruses and also provided an in-depth understanding of how different viruses subvert phase-specific subunits to manipulate the same periodic complex, which is exemplified by the cyclin B-CDK1 complex (complex ID = 2452; Fig. 5C). The cyclin B-CDK1 complex is mainly constituted by CDK kinase (CDK1), cyclin proteins (CCNB1 and CCNA2), CDK inhibitors (CDKN1B and CDKN1C), and CDK regulatory protein (CDKS1B). The kinase activity of CDK1 can be activated by the cyclin proteins or inhibited by the CDK inhibitors. Previous studies have shown that the activity of cyclin B-CDK1 can be inhibited by multiple viruses, such as HIV-1 and HPV, to prevent the host cell from entering mitosis (M phase) (39, 40). By combining viral targeting and periodic information of subunits within complexes, we observed that H1N1, EBV, and HCV target CDK1 (which is highly expressed in the G_2_-M phase), HPV targets CDKN1B (which is highly expressed in the S-G_2_ phase), and HIV-1 targets both CDK1 and CDKN1B. In addition to the differences in targeted proteins, temporal differences in the expression of targeted proteins indicated that different viruses might manipulate the same complex at different cell cycle phases. Moreover, our temporal mapping of VTCs during the host cell cycle has identified a number of periodic complex candidates whose functional roles during viral infection remain elusive. Undoubtedly, these periodic complexes will provide important hints for conducting hypothesis-driven functional interrogation experiments.

As a potential application of VTC-based analysis, we proposed a VTC-based strategy in prioritizing drug targets. Due to the complexity of drug target discovery, the computational discovery of antiviral druggable targets is mainly through integrating different information (48). For example, Watanabe et al. combined the results of a large-scale RNAi assay and a host-influenza virus interactome to select viral targets as potential drug targets (22). Cheng et al. used a systems biology-based method by integrating gene-trap insertional mutagenesis with a host-virus interactome to identify putative druggable antiviral targets (49). Note that the principle of antiviral drug target discovery in the above two studies is focused on the intersection of viral targets and HDFs. Similarly, our identification of drug targets also considered information about viral targets and HDFs. Moreover, we also integrated other heterogeneous information, such as the expression of druggable targets in virally targeted tissues and innate immune-related proteins. We aimed to identify drug targets that were vital for maintaining the function of complexes. By adopting a biologically driven strategy, we hope that the identified druggable targets in this work are worthy of further experimental verification.

In general, the current understanding of VTCs is far from complete, and there are still some limitations to this work. For instance, some inconsistent observations among the different viruses should be further investigated with the increasingly available human-virus PPI data. It is also worth mentioning that our computational framework under investigation is protein-centric. To further examine the dynamic response of VTCs under viral infection, an in-depth integration of diverse transcriptional and proteomic data obtained during viral infection will be highly beneficial. Regarding the future, some interesting questions need to be answered. In this work, we found that viruses targeted subunits with higher within-complex degrees. Are these viral targets located in the geometric centers of the complexes? How do human-virus interactions affect the process of complex assembly? With the growing number of studies on the 3D structures and assembly of protein complexes (50), we can analyze the 3D structural features of viral targets within complexes, which can allow us to have a deeper understanding of the molecular principles that underline the hijacking of host protein complexes by viruses. Moreover, we observed that housekeeping proteins, HDFs, and innate immune proteins tended to appear in VTCs, but what are the functional roles of these overlapping complexes and how do these proteins interact with each other within complexes? Experimental and bioinformatics scientists should work together to accelerate the elucidation of molecular interaction mechanisms.

In summary, we systematically identified the VTCs of five viruses (H1N1, HIV-1, EBV, HPV, and HCV) and conducted a comprehensive analysis of VTCs. Regarding the topology and functional features of viral targets in the context of VTCs, we found that viral targets tended to have a high within-complex degree, and they also tended to be scaffold proteins and housekeeping proteins. These differences between targets and nontargets shed light on the potential molecular principles utilized by viruses to hijack human protein complexes. In terms of functional distribution of VTCs, we found that complexes essential for viral propagation were simultaneously targeted by multiple viruses. We characterized the dynamic expression of subunits within VTCs during the host cell cycle, which reflects how viruses manipulate host protein complex assembly. Finally, an online platform for VTCs and relevant information of complex-based antiviral drug discovery was developed. Overall, the current comprehensive analysis of VTCs increases our understanding of the mechanisms of viral infection at the protein complex level, and we hope VTcomplex will become a useful data resource for the community.

## MATERIALS AND METHODS

### Data sets.

**(i) Human protein complexes and PPIs.** A total of 4,659 protein complexes were originally obtained from hu.MAP (http://proteincomplexes.org ), which were named by digital IDs from 0 to 4658. Protein subunits with unreviewed UniProt IDs were filtered. Complexes containing less than two subunits were further removed. Thus, 4,588 complexes were retained. Experimentally validated human PPIs were collected from BioGRID (51), IntAct (52), and DIP (53). After removing the redundant and genetic interactions, we obtained 17,079 unique PPIs among proteins within these 4,588 protein complexes.

**(ii) Human-virus PPIs.** We first collected experimentally determined human-virus PPIs from HPIDB 2.0 and further removed PPIs containing proteins without UniProt IDs. Our analysis covered five common human-pathogenic viruses, including two RNA viruses (H1N1 and HIV-1) and three DNA viruses (EBV, HPV, and HCV). In line with previous studies (15, 16), we further integrated PPIs that involved in different strains of the same virus.

**(iii) Tissue-specific expression data, housekeeping genes, and essential genes.** The tissue-specific expression values of human genes were obtained from the GTEx Portal, which collects gene expression information for 53 human tissues (20). In this work, genes with a TPM (transcripts per million) of >1 for any tissue in GTEx were defined as housekeeping genes, which is similar to the definition used for the Human Protein Atlas database (54). Thus, 7,956 housekeeping genes were identified. In addition, 1,731 essential genes that are indispensable for cell survival were collected from Blomen et al. (55).

**(iv) Scaffold proteins, HIV-1’s HDFs, and human innate immune-related proteins.** We collected 276 human scaffold proteins from ScaPD (56), 850 HDFs related to HIV-1 from three large-scale loss-of-function screening studies (33, 57, 58), and 1,353 innate immune-related proteins from InnateDB (59).

### Prioritization of VTCs for each virus.

Taking HIV-1 as an example, we first conducted Fisher’s exact test to calculate the statistical significance (*P* value) corresponding to a complex targeted by HIV-1. Then, the *P* value after the Benjamini-Hochberg correction, which was defined as VT_significance_, was used to measure the extent of the complex that was targeted by HIV-1. In general, VTCs for which VT_significance_ < 0.05 were considered to be significantly targeted by HIV-1. Note that prioritization of VTCs was conducted for each virus.

### Annotation of human protein complexes targeted by viruses.

To annotate each protein complex, we used g:Profiler (60) to conduct enrichment analysis, which covers gene ontology, biological pathways, and protein complexes. The reference complex database was CORUM. Enriched terms with corrected *P* values of <0.05 were retained, and all the results are available in Data Set S2 in the supplemental material. In addition to assigning a unique digital ID, we named each complex based on two rules. First, for those complexes with at least one enriched term in CORUM, we used the most significantly enriched term as the complex name, which was similar to a previous study (61). Second, the complexes without enriched terms in CORUM were named by the corresponding subunits’ gene names connected by hyphens. The second rule was also adopted for the CORUM database. Note that the above naming strategy was not entirely precise, but it generally allowed us to summarize the biological functionalities of the complexes. To ensure reliability, we also conducted manual correction for some of the complexes mentioned in this work through other enrichment information.

### Calculation of the within-complex degree.

Taking each VTC as a subnetwork, we used igraph (https://igraph.org/) to calculate the within-complex degree for each subunit. Only those VTCs that simultaneously met the following criteria were taken into account. (i) The complexes contained more than two subunits. (ii) The complexes contained both viral targets and nontargets simultaneously. (iii) Both targets and nontargets had at least one interaction. To facilitate the comparison of different VTCs, the within-complex degree of each subunit was normalized by a Z-score within a VTC.

### d_N_/d_S_ measurement.

The d_N_/d_S_ value is often used to measure the strength of natural selection of a gene or protein. A larger d_N_/d_S_ value corresponds to a higher evolutionary rate. To obtain the evolutionary rate of human proteins, we used the mouse as the reference species. All d_N_/d_S_ values were calculated using http://asia.ensembl.org/biomart/martview/ based on the human genome version GRCh38.

### Identification of periodic complexes.

Periodic genes were collected from Cyclebase 3.0 (62) and a recently published high-resolution transcriptome of the human cell cycle (21). Based on the time point of peak expression in the transcriptome experiment (21), the 1,221 periodic genes obtained were further assigned to six cell cycle phases (G_1_, G_1_-S, S, S-G_2_, G_2_-M, and M-G_1_). Similar to the definition of DEC, a complex with at least one periodic protein was defined as periodic. Moreover, we assigned each periodic complex to a specific periodic phase. Taking the G_1_ phase as an example, a periodic complex assigned to this phase should meet one of the following two criteria. (i) All periodic proteins within the complex are assigned to G_1_. (ii) The number of periodic proteins present in G_1_ is greater than twice the sum of the periodic proteins present in other phases. Otherwise, the complex was classified as Mixed. All periodic proteins and classified periodic complexes are listed in Data Set S3.

### Gene expression specificity of drug targets between HIV-1-targeted tissues and other tissues

Although HIV-1 infection is cell specific, we used tissues that were most enriched for T cells as the targeted tissues to characterize this specificity. Based on GTEx’s tissue categories, we defined blood, lymphocytes, and spleen as HIV-1-targeted tissues. We used DT_specificity_ to measure the specificity of the drug target between HIV-1-targeted tissues and other tissues, which was calculated by dividing the mean gene expression value in the targeted tissue by the mean expression value in other tissues.

### ID mapping.

The online UniProt ID mapping tool (https://www.uniprot.org/uploadlists/) was used to match all of the human or viral gene IDs to UniProt IDs.
