# Supplementary figures and images for: Understanding Human-Virus Protein-Protein Interactions Using a Human Protein Complex-Based Analysis Framework
Source: mSystems. 2019 Apr 9;4(2):e00303-18. doi: 10.1128/mSystems.00303-18 (PMC6456672; doi:10.1128/mSystems.00303-18)

A

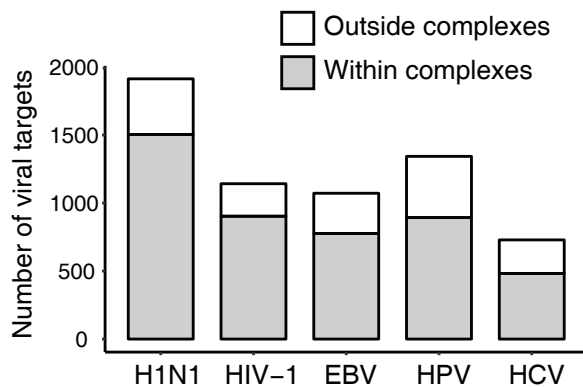

B

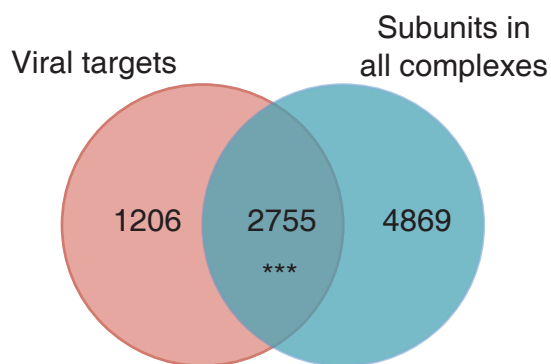

Supplement: FIG S1 [file mSystems.00303-18-sf001.pdf]

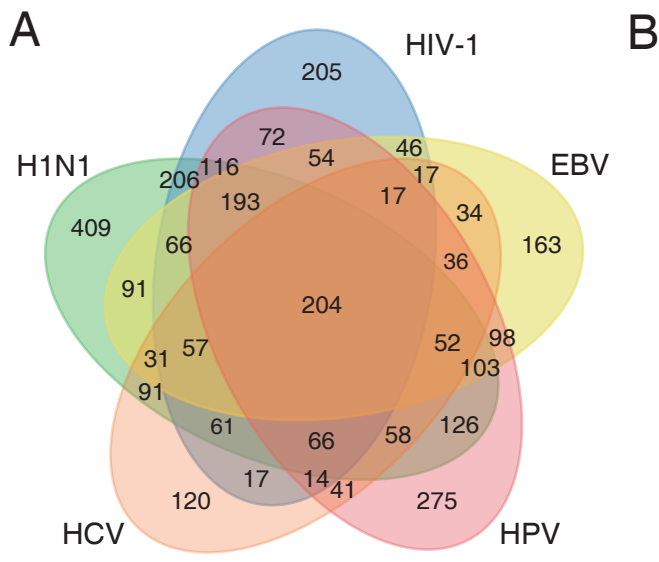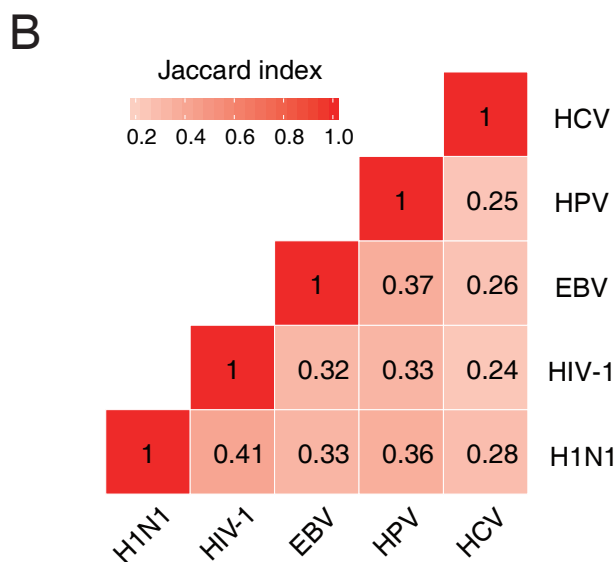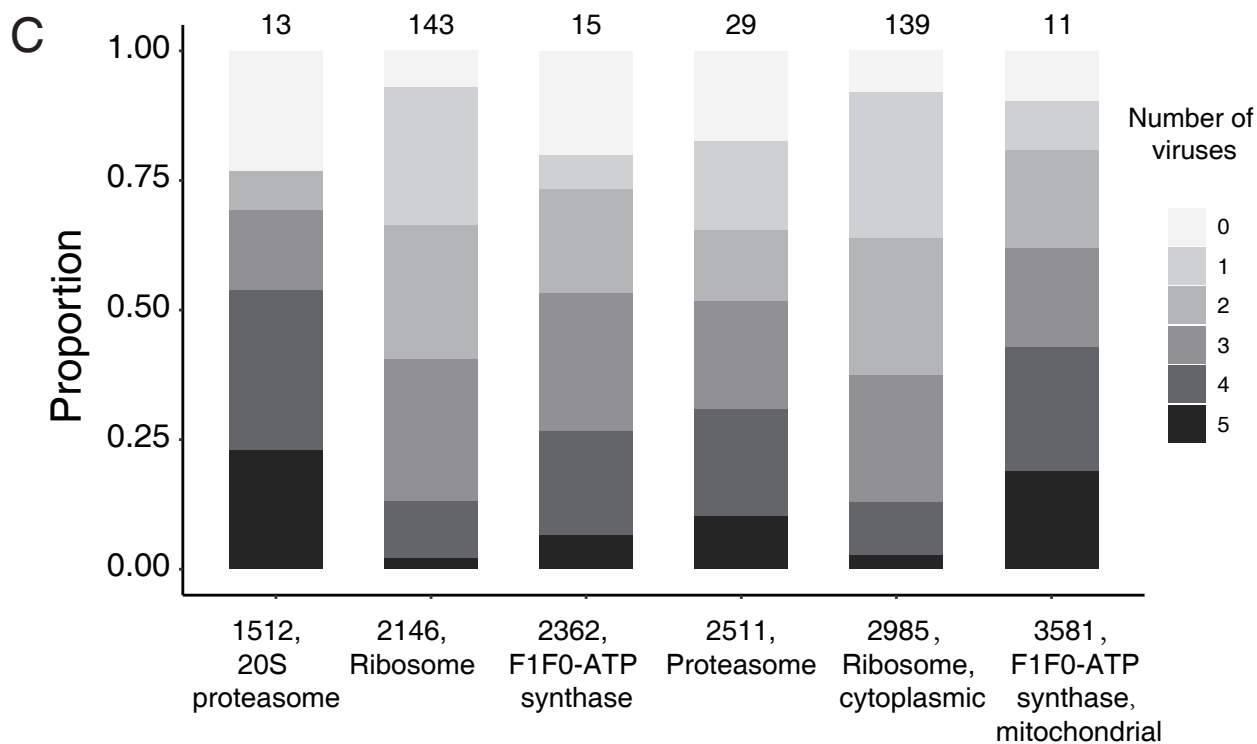

Supplement: FIG S2 [file mSystems.00303-18-sf002.pdf]

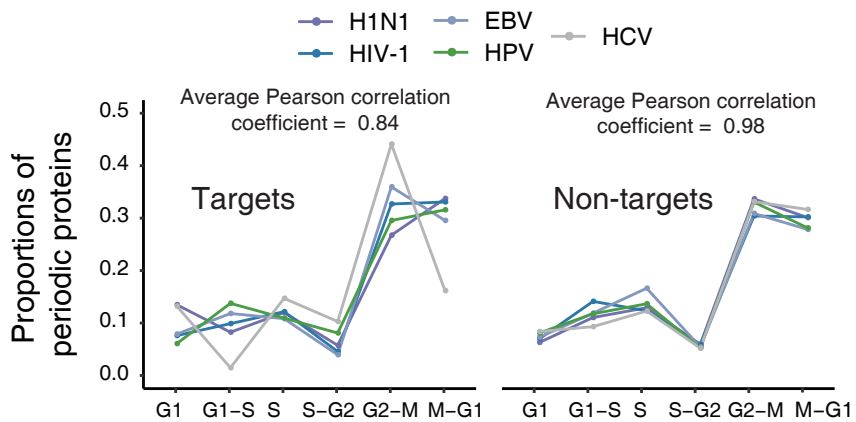

Supplement: FIG S3 [file mSystems.00303-18-sf003.pdf]

## H1N1

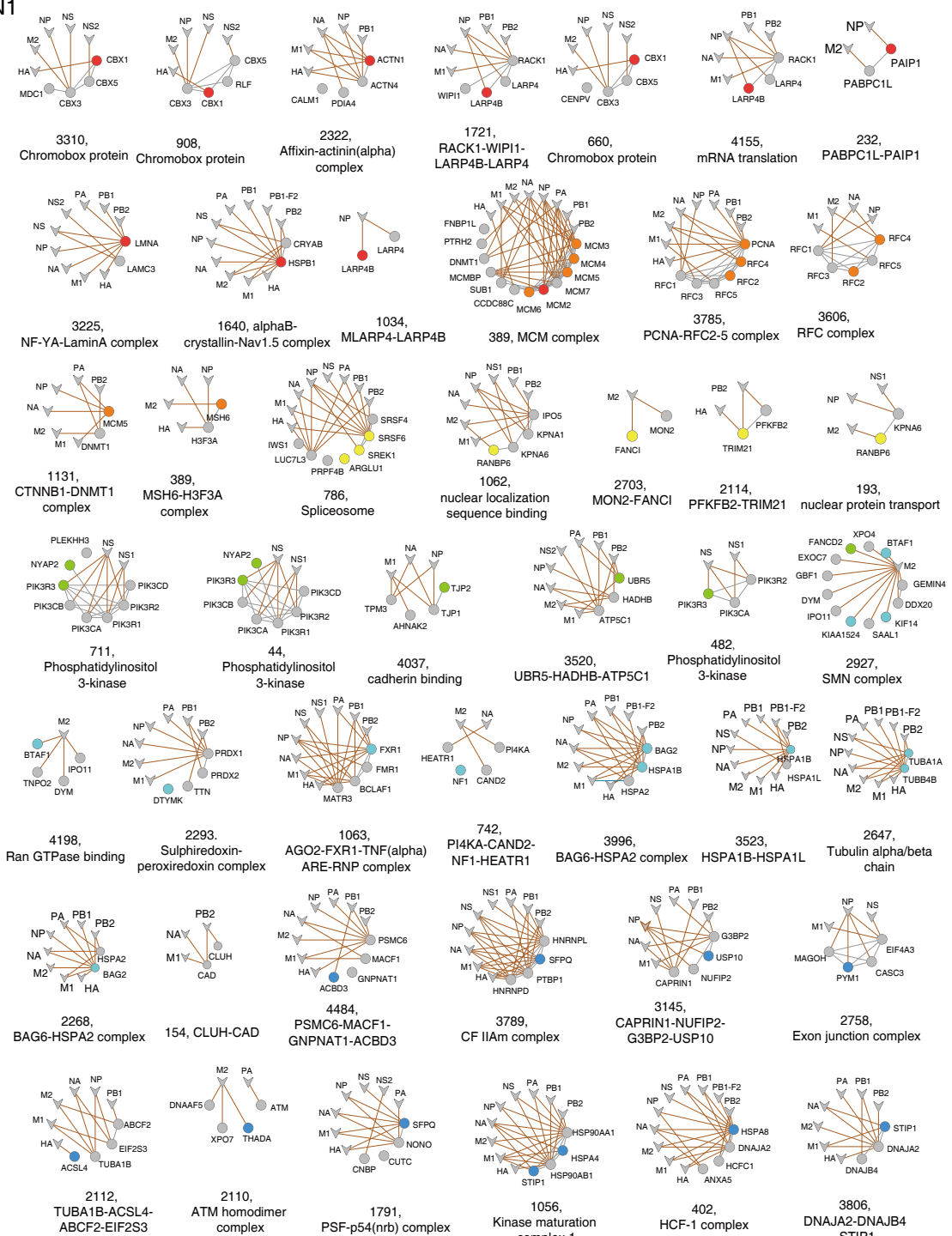

## HIV-1

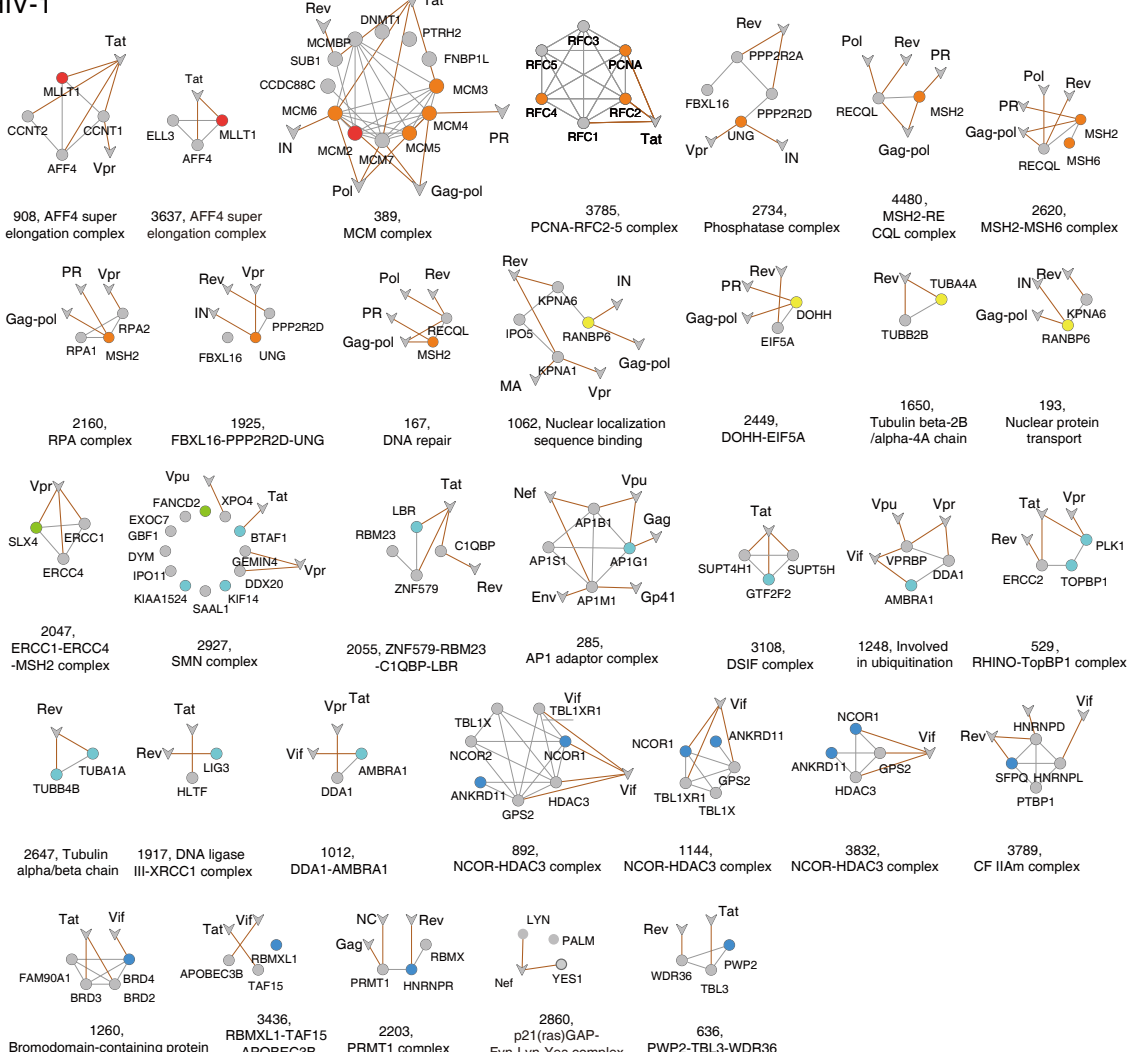

## EBV

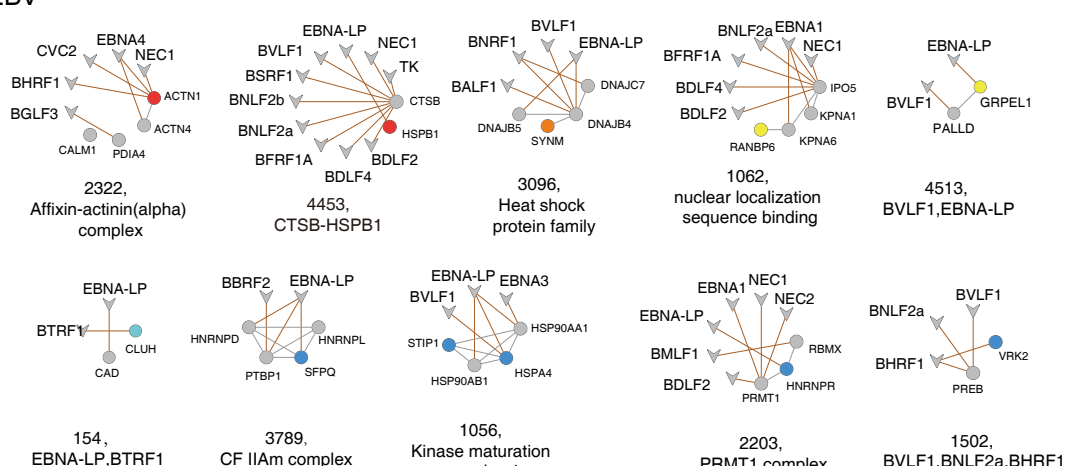

## HPV

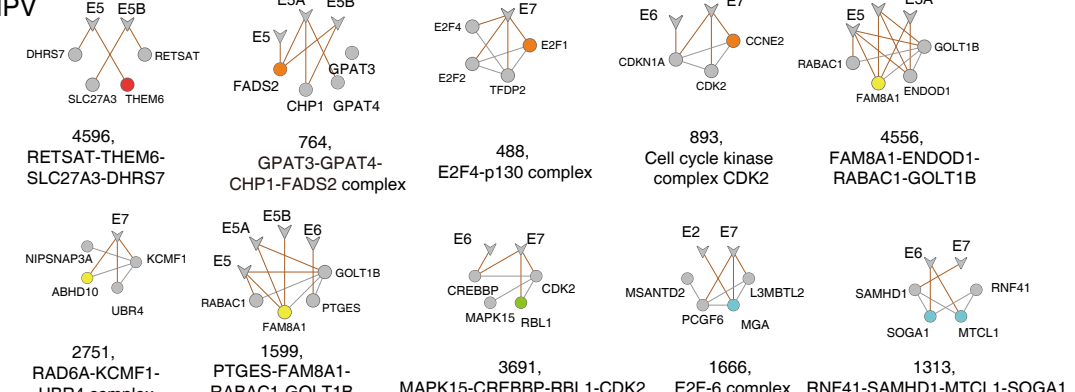

## HCV

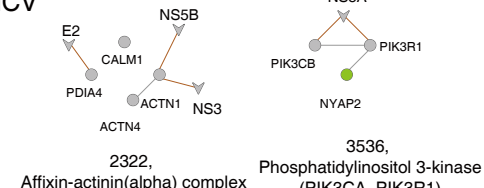

Supplement: FIG S4 [file mSystems.00303-18-sf004.pdf]

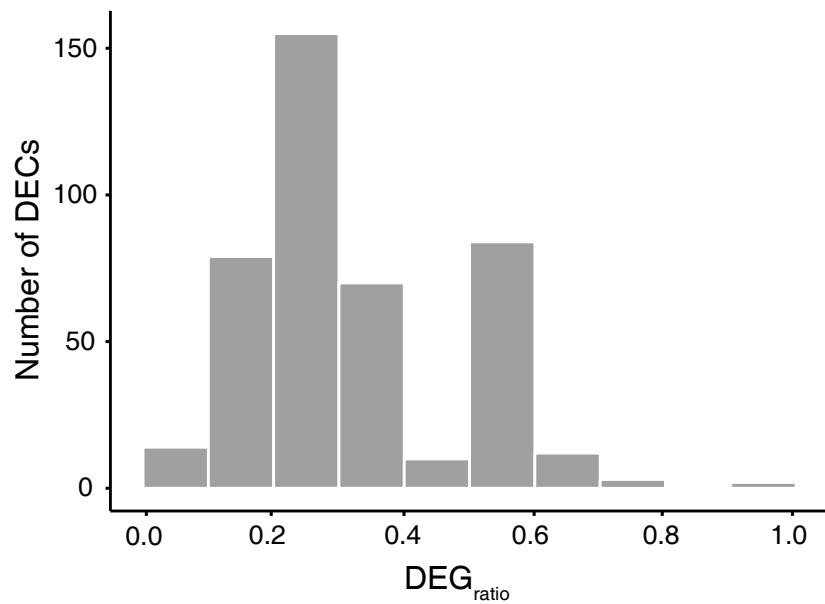

Supplement: FIG S5 [file mSystems.00303-18-sf005.pdf]
