# Supplementary material for: Understanding Human-Virus Protein-Protein Interactions Using a Human Protein Complex-Based Analysis Framework
Source: mSystems. 2019 Apr 9;4(2):e00303-18. doi: 10.1128/mSystems.00303-18 (PMC6456672; doi:10.1128/mSystems.00303-18)
Supplement: TABLE S1 [file mSystems.00303-18-st001.docx]

**Table S1.** The PubMed IDs used for counting the Jaccard index at the target/VTC level.

| **Viral name** | **PubMed ID 1** | **PubMed ID 2** | **Jaccard index** | **Level** |
| --- | --- | --- | --- | --- |
| H1N1 | 25464832 | 26651948 | 0.147 | Target |
| H1N1 | 25464832 | 26651948 | 0.320 | VTC |
| HIV-1 | 22190034 | 22174317 | 0.033 | Target |
| HIV-1 | 22190034 | 22174317 | 0.129 | VTC |
| EBV | 22810586 | 22761553 | 0.041 | Target |
| EBV | 22810586 | 22761553 | 0.111 | VTC |
| HCV | 18985028 | 25616068 | 0.006 | Target |
| HCV | 18985028 | 25616068 | 0.055 | VTC |
| HPV | 22810586 | 22898364 | 0.032 | Target |
| HPV | 22810586 | 22898364 | 0.116 | VTC |
