# Supplementary material for: Understanding Human-Virus Protein-Protein Interactions Using a Human Protein Complex-Based Analysis Framework
Source: mSystems. 2019 Apr 9;4(2):e00303-18. doi: 10.1128/mSystems.00303-18 (PMC6456672; doi:10.1128/mSystems.00303-18)
Supplement: FIG S6 [file mSystems.00303-18-sf006.pdf]

① Input keywords

UniProt ID, protein/gene name, complex name/ID are supported.

Protein/Gene name ▼ | Input search keywords | Search | Reset

② Choose an interested virally-targeted complexes

Information about the query protein/gene and virally-targeted complexes are presented.

UniProtKB	Protein names	Gene names	Virus	Complex id	Complex name	Complex size	Target ratio	P-value	Q-value	Details
P12504	Virion infectivity factor	Vif	HIV-1	1663	BAG6-HSPA2 complex	5	0.80 (4/5)	4.84E-05	1.16E-03	View
P12504	Virion infectivity factor	Vif	HIV-1	646	Cullin-RING E3 ubiquitin ligase complex	4	0.75 (3/4)	6.89E-04	7.36E-03	View
P12504	Virion infectivity factor	Vif	HIV-1	779	Cullin-RING E3 ubiquitin ligase complex	4	0.75 (3/4)	6.89E-04	7.36E-03	View
P12504	Virion infectivity factor	Vif	HIV-1	2339	KPNA4-HUWE1-KPNA3	3	0.67 (2/3)	9.20E-03	4.71E-02	View
P12504	Virion infectivity factor	Vif	HIV-1	534	DDB2 complex	21	0.62 (13/21)	7.42E-12	1.16E-09	View
P12504	Virion infectivity factor	Vif	HIV-1	4442	Ubiquitin E3 ligase (CSN1, CSN8, HRT1, SKP1, SKP2, CUL1, CUL2, CUL3)	8	0.38 (3/8)	8.12E-03	4.71E-02	View
P12504	Virion infectivity factor	Vif	HIV-1	458	KPNA4-BCL11A-NRDC-HUWE1-KPNA3	5	0.60 (3/5)	1.65E-03	1.50E-02	View
P12504	Virion infectivity factor	Vif	HIV-1	2846	PA28gamma complex	3	0.33 (1/3)	1.60E-01	2.44E-01	View
P12504	Virion infectivity factor	Vif	HIV-1	1241	PA28gamma complex	6	0.17 (1/6)	2.95E-01	3.16E-01	View
P12504	Virion infectivity factor	Vif	HIV-1	1782	DNAJB6-DNAJB8-DNAJB2	3	0.33 (1/3)	1.60E-01	2.44E-01	View

Showing 1 to 10 of 101 entries

Previous 1 2 3 4 5 … 11 Next

③ Download information about virally-targeted complexes

Information such as host dependency factors, innate immune-related proteins and potential drug targets and drugs are presented.

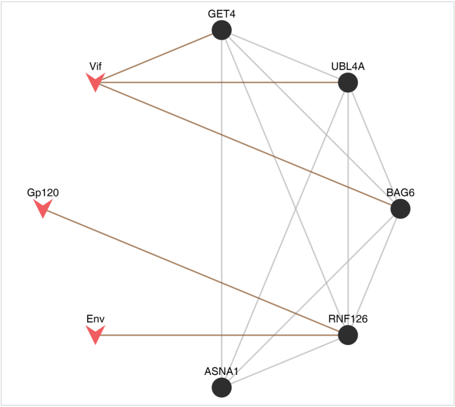

Save Image As JPG | Save Image As Text

Detailed information about virally-targeted complex

Complex ID: 1663

Complex name: BAG6-HSPA2 complex

Targeted by virus: HIV-1

virally-targeted significance: 1.16E-03

Targeted by other viruses: H1N1 EBV HCV

Number of targets/Complex size: 4/5

Complex subunits: GET4 UBL4A BAG6 RNF126 ASNA1

Host dependent factors: BAG6

Differential expression genes: NA

Innate immune-related proteins: NA

Potential drug targets: ASNA1

Potential drug-target interactions: DB00171-ASNA1

Steps for using VTcomplex
